# Serum microRNA expression quantitative trait loci in children with asthma colocalize with asthma-related GWAS results

**DOI:** 10.1038/s41525-025-00510-7

**Published:** 2025-07-17

**Authors:** Julian Hecker, Anshul Tiwari, Rinku Sharma, Kevin Mendez, Jiang Li, Sofina Begum, Qingwen Chen, Albert Smith, Juan C. Celedón, Scott T. Weiss, Rachel S. Kelly, Jessica A. Lasky-Su, Kelan G. Tantisira, Michael McGeachie

**Affiliations:** 1 Channing Division of Network Medicine, Department of Medicine, Brigham and Women’s Hospital and Harvard Medical School, Boston, MA USA; 2 Department of Molecular Physiology and Biophysics, Vanderbilt University, Nashville, TN USA; 3 Clinical Big Data Research Center, The Seventh Affiliated Hospital of Sun Yat-sen University, Shenzhen, China; 4 Shenzhen Key Laboratory of Chinese Medicine Active Substance Screening and Translational Research, Shenzhen, China; 5 Center for Statistical Genetics, Department of Biostatistics, University of Michigan School of Public Health, Ann Arbor, MI USA; 6 Division of Pulmonary Medicine, Department of Pediatrics, UPMC Children’s Hospital of Pittsburgh, University of Pittsburgh, Pittsburgh, PA USA; 7 Division of Pediatric Respiratory Medicine, Department of Pediatrics, University of California San Diego, San Diego, CA USA; 8 Division of Pediatric Respiratory Medicine, Department of Pediatrics, Rady Children’s Hospital of San Diego, San Diego, CA USA

**Keywords:** Quantitative trait loci, Asthma

## Abstract

Asthma poses a significant public health burden. Despite identifying more than a hundred genetic risk loci in genome-wide association studies (GWAS), the underlying functional mechanisms remain poorly understood. Studying omics, especially microRNAs (miRNAs), is a promising approach to facilitate our understanding of the biological pathways of asthma. Here, we performed miRNA expression quantitative trait loci (miRNA-QTL) analyses using whole-genome sequencing and serum-based miRNA expression data from two independent cohorts of children with asthma (Genetic Epidemiology of Asthma in Costa Rica Study (GACRS), (NCT00021840, 2005-06-23) (*N* = 980, Discovery) and the Childhood Asthma Management Program (CAMP) (NCT00000575, 2005-06-23) (*N* = 354, Replication)). Our robust discovery analysis identified 28 significant cis-miRNA-QTL associations, where 12 were not reported in three independent miRNA-QTL studies. Three of these 12 signals were replicated in CAMP. The QTLs colocalize with expression and splicing QTL in asthma-relevant tissues and cells, and overlap with asthma-related and blood cell trait GWAS hits.

## Introduction

Asthma is a common chronic respiratory disease that affects more than 300 million people worldwide and imposes a significant global health burden^1–4^. Although recent large-scale genome-wide association studies (GWAS) identified more than a hundred genetic associations with asthma^5,6^, the underlying functional mechanisms of asthma remain poorly understood and are hypothesized to comprise a complex interplay between genetic, early life, and environmental factors^7^. Moreover, asthma is considered an umbrella diagnosis covering several phenotypes with distinct biological pathways and clinical manifestations^8,9^, increasing the complexity of understanding and studying the etiology.

Multiple omics levels have been studied to disentangle the biological pathways of asthma, including genomics, metabolomics, epigenomics, and transcriptomics. Recently, non-coding RNAs, specifically microRNAs (miRNAs), and their effects on the immune system have attracted more attention in asthma research^10^. MiRNAs are small non-coding RNA molecules ranging from 18 and 22 nucleotides acting as post-transcriptional regulators of target gene expression by interacting with messenger RNA and playing a significant role in regulating epithelial cell and inflammatory processes^11^. These processes can contribute to airway remodeling and obstruction^10,12^ and implicate miRNAs as essential biomarkers and research targets for understanding asthma development. Furthermore, miRNAs are gaining attention as a therapeutic tool to restore cell functions that are altered in the development of diseases^13^.

Previous work demonstrated that genetic variants regulate the expression levels of miRNAs in plasma and blood^14,15^. These genetic variants are referred to as miRNA expression quantitative trait loci (miRNA-QTLs). The identification of miRNA-QTLs has the potential to link the genetic factors associated with asthma and related traits to biological mechanisms through miRNAs.

In this study, we sought to identify serum miRNA-QTLs in children with asthma. Our analyses utilized whole-genome sequencing (WGS) data for the two family-based childhood asthma cohorts ‘Genetic Epidemiology of Asthma in Costa Rica Study’ (GACRS)^16^ and the Childhood Asthma Management Program (CAMP)^17^. We performed cis- and trans-analyses using a discovery and replication approach. The identified miRNA-QTLs were further investigated for colocalization with expression QTLs, splicing QTLs, and asthma-related GWAS results.

## Results

The workflow of our analyses is visualized in Supplementary Fig. 1.

### Study populations with WGS and miRNA data

After quality control, we had 980 GACRS offspring and 354 CAMP offspring samples with both WGS and miRNA expression data at our disposal. The baseline characteristics of both study cohorts are described in Table 1. The general baseline factors were comparable between GACRS and CAMP, except for race/ethnicity and lung function. CAMP subjects are ethnically/racially diverse and had slightly lower lung function measurements, while GACRS subjects are of Hispanic (Costa Rican) ancestry. The total number of miRNAs in GACRS and CAMP successfully mapped to genomic positions after quality control was 316 and 264, respectively. The overlap consisted of 252 miRNAs. The number of cis-miRNA windows in the miRNA-QTL analyses was 361 in GACRS and 303 in CAMP, reflecting the mapping of miRNAs to multiple genomic positions as obtained based on recent miRbase (v22) data. A total of 5,872,254 and 5,295,172 biallelic common autosomal genetic variants were available for analysis in GACRS and CAMP after quality control, respectively.

**Table 1:** Characteristics of the children with asthma included in the analysis

	GACRS	CAMP	*p*-value
N	980	354	
Sex (female %)	40%	41%	*p* = 0.87*
Age (mean (sd) in years)	9.25 (1.87)	8.99 (2.17)	*p* = 0.05**
Height (mean (sd) in meters)	1.33 (0.12)	1.34 (0.14)	*p* = 0.10**
FEV_1_ (mean (sd) in liter)	1.78 (0.50)	1.65 (0.46)	*p* = 2.00×10^-5^**
FEV_1_% predicted (mean (sd))	98.63 (17.08)	93.29 (13.06)	*p* = 2.64×10^-9^**
Maternal asthma history (yes/no/unknown)	298/678/4 (30%/69%/1%)	98/249/7 (28%/70%/2%)	*p* = 0.46*
Paternal asthma history (yes/no/unknown)	253/716/11 (26%/73%/1%)	72/261/21 (20%/74%/6%)	*p* = 0.12*
Ethnicity	Hispanic (100%)	280 (79%) European American/ 58 (16%) African American/ 16 (5%) Hispanic	*p* < 10^−16^*

*based on chi-squared-test.

**based on *t* test.

### Cis-miRNA QTL analysis

**Fig. 1:**
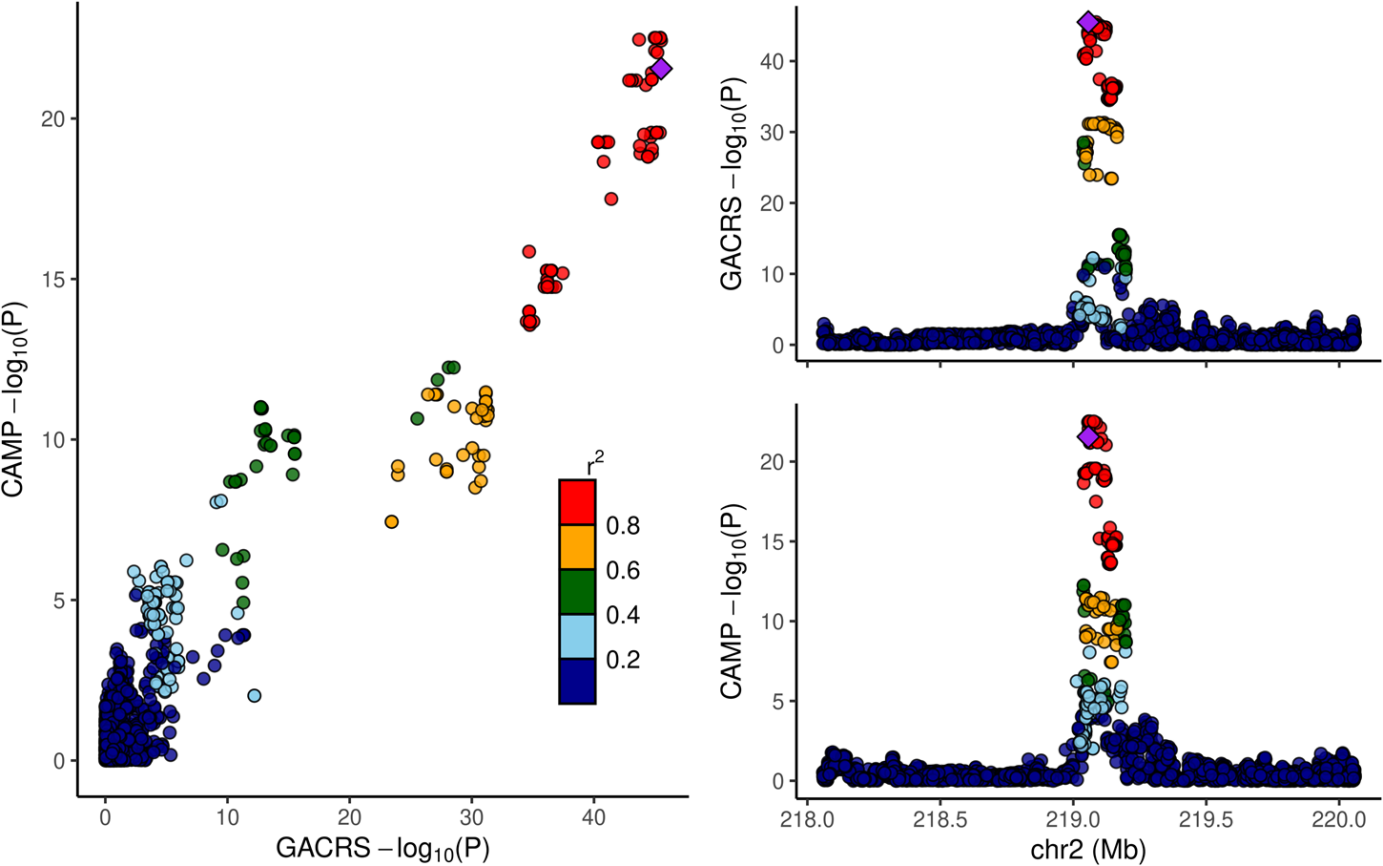
LocusCompare plot of cis-miRNA-QTL statistics for hsa-miR-3131 in GACRS and CAMP data. -log10(P) denotes the negative logarithm of the nominal association *p*-values. ${r}^{2}$Values report the strength of linkage disequilibrium between the lead variant (purple) and the corresponding other genetic variant; colors indicate the strength of linkage disequilibrium. This plot was created using the *LocusCompareR* R package.

The GACRS cis-analysis incorporated 321 cis-miRNA windows with overlapping genetic variants. We identified a total of 28 top-cis-miRNA-QTLs in GACRS with an FDR *q*-value q<=0.05 (Table 2) using tensorQTL^18^. The corresponding miRNAs are located across different chromosomes, but notably, 11 miRNAs are on chromosome 14 in the 14q32 cluster^19^. For the replication analysis, 22 of the 28 implicated miRNAs were available in the CAMP analysis. In particular, 20 of the 28 variant-miRNA pairs were directly available in CAMP for replication analysis, and one additional variant-miRNA pair was tested using a proxy SNP in high LD (for hsa-miR-181a-5p, we tested rs2360962 in CAMP instead of rs7529925). One of the 22 miRNAs had no information regarding the implicated genetic variants available in CAMP (hsa-miR-3679-5p, top cis-miRNA QTL and LD proxies had MAF < 5% in CAMP) and was, therefore, not considered in the replication analysis. We observed that 7 of these pairs had a nominally significant association *p*-value p< =0.05 in CAMP. For four pairs, the *p*-value was significant after correcting for 21 tests using a Bonferroni correction at the significance level of 0.05, with a consistent direction of effect. The corresponding miRNAs were hsa-miR-4433b-3p, hsa-miR-3131, hsa-miR-181a-5p, and hsa-miR-335-3p. Here, hsa-miR-3131 showed strong genetic associations in both GACRS and CAMP, and a colocalization analysis between both cis-QTL statistics resulted in a high posterior probability of a consistent signal (colocalization posterior probability > =0.95), visualized in Fig. 1. The relationship between the top-cis-miRNA-QTL, rs6751804, and hsa-miR-3131 expression is illustrated in Supplementary Fig. 2. We also observed strong evidence for a shared genetic factor for the miRNAs hsa-miR-127-3p, hsa-miR-136-3p, hsa-miR-370-3p, and hsa-miR-409-3p on 14q32, displayed in Supplementary Figs. 3 and 4 (colocalization posterior probability 0.89). As a sensitivity analysis, we investigated the differences between the corresponding nominal association *p*-values for the 28 top-cis-miRNA-QTL signals when incorporating the top five or top ten miRNA expression principal components derived from the ReFACTor approach^20^ (see Methods). The results remained qualitatively unchanged. The same was observed when replacing the principal components with the factors derived from the non-negative matrix factorization (see Methods). Additional sensitivity analyses stratified by age group (<=9 years of age vs. > 9 years of age) showed no significant differences in both GACRS and CAMP after Bonferroni correction. This also holds true for interaction analyses between QTL and the first principal component of genetic ancestry. The 28 cis-miRNA-QTL associations are described in full detail in Supplementary Table 1. Furthermore, all 28 miRNAs were found to be expressed in lung and lung bronchus tissue (Supplementary Table 6) in the miRNATissueAtlas2^21^. Finally, we performed a meta-analysis based on the cis-miRNA-QTL statistics between GACRS and CAMP, for all overlapping miRNAs. The results of this meta-analysis did not yield any additional findings.

**Table 2:** Significant top-cis-miRNA-QTL results in GACRS (FDR *q*-value < =0.05)

miRNA	CHR	variant	AF	*p*-value nominal	*q*-value	replication CAMP	replication Nikpay et al.	replication Huan et al.	replication Mustafa et al.
hsa-miR-181a-5p	1	rs7529925	0.72	4.65E−10	1.50E−04	**Yes**	**Yes**	**Yes**	**Yes**
hsa-miR-4433b-3p	2	rs75982381	0.18	5.47E−14	4.92E−08	**Yes**	NA	No	NA
hsa-miR-3679-5p	2	rs10175383	0.11	2.24E−13	2.00E−07	NA	No	No	No
hsa-miR-3131	2	rs6751804	0.52	3.21E−46	6.55E−36	**Yes**	No	No	No
hsa-miR-577	4	rs35736852	0.12	2.84E−27	4.33E−19	NA	No	No	No
hsa-miR-584-5p	5	rs36047	0.49	1.58E−07	1.30E−02	No	**Yes**	No	**Yes**
hsa-miR-550a-3-5p	7	rs1230047	0.39	5.91E−07	1.36E−02	NA	No	No	**Yes**
hsa-miR-335-3p	7	rs79257663	0.18	1.72E−11	7.52E−06	**Yes**	No	No	No
hsa-miR-873-3p	9	rs1368998	0.10	3.53E−08	1.52E−03	No	No	No	No
hsa-miR-873-5p	9	rs2211382	0.30	1.04E−07	3.59E−03	NA	No	No	**Yes**
hsa-miR-1908-5p	11	rs174556	0.43	9.86E−10	9.95E−05	No	**Yes**	No	**Yes**
hsa-miR-100-5p	11	rs140471379	0.37	3.85E−11	9.88E−06	No	**Yes**	No	No
hsa-miR-625-5p	14	rs35287494	0.05	5.31E−06	4.85E−02	NA	No	No	No
hsa-miR-625-3p	14	rs2127870	0.78	1.37E−08	7.95E−04	No	NA	**Yes**	NA
hsa-miR-493-5p	14	rs61993261	0.25	4.61E−07	1.27E−02	NA	No	No	No
hsa-miR-127-3p	14	rs4900471	0.50	2.06E−08	3.03E−03	No	**Yes**	**Yes**	**Yes**
hsa-miR-432-5p	14	rs12588718	0.47	2.04E−06	3.52E−02	No	No	No	**Yes**
hsa-miR-136-3p	14	rs8012970	0.42	5.70E−08	2.57E−03	No	No	No	**Yes**
hsa-miR-370-3p	14	rs12882815	0.53	1.97E−07	7.35E−03	No	No	No	**Yes**
hsa-miR-411-3p	14	rs12432955	0.75	6.67E−07	1.46E−02	NA	No	**Yes**	**Yes**
hsa-miR-654-5p	14	rs35638446	0.23	2.58E−06	3.37E−02	No	No	No	No
hsa-miR-382-5p	14	rs8012970	0.42	3.12E−06	4.26E−02	No	No	**Yes**	**Yes**
hsa-miR-323b-3p	14	rs6575812	0.32	9.76E−12	6.87E−06	No	**Yes**	No	**Yes**
hsa-miR-409-5p	14	rs4243729	0.46	1.65E−06	3.34E−02	No	No	No	No
hsa-miR-409-3p	14	rs12881760	0.41	1.07E−06	3.34E−02	No	No	**Yes**	**Yes**
hsa-miR-3615	17	rs9909546	0.07	1.19E−06	2.65E−02	No	NA	No	NA
hsa-miR-1-3p	20	rs60640728	0.20	3.46E−11	1.25E−05	No	NA	No	NA
hsa-miR-941	20	rs2427555	0.44	9.41E−11	3.21E−05	No	**Yes**	**Yes**	**Yes**

Replication information for CAMP, Nikpay et al.^14^, Huan et al.^15^, and Mustafa et al.^22^ data are shown in the last four columns. NA: miRNA/proxy variants not available/not included in corresponding study. For the Huan et al. study, ‘no’ reflects either non-available miRNAs or no replication.

*AF* allele frequency, *FDR* False Discovery Rate, *CHR* Chromosome.

### Trans-miRNA QTL analysis

A trans-analysis using tensorQTL identified three significant trans-associations involving the miRNAs hsa-miR-148a-3p, hsa-let-7g-5p, and hsa-miR-15a-5p. These associations did not replicate in CAMP. Notably, all corresponding genetic variants in the GACRS analysis are low-frequency variants. The results are reported in Supplementary Table 2; further research is required to confirm or refute the observed associations.

### Replication using results from previously published miRNA-QTL analyses

We investigated whether the significant cis-miRNA-QTL associations were previously reported in three external miRNA-QTL studies.

Overall, 24 of the 28 miRNAs implicated by our observed cis-miRNA-QTL associations were also investigated by Nikpay et al.^14^ A total of 7 cis-miRNA-QTL associations showed evidence for replication (Table 2). The results for hsa-miR-181a-5p, hsa-miR-584-5p, hsa-miR-1908-5p, hsa-miR-323b-3p, and hsa-miR-941, demonstrated replication based on the variant-miRNA association *p*-value (directly or using a proxy genetic variant) and showed evidence for colocalization (colocalization posterior probability > =0.8). For hsa-miR-100-5p and hsa-miR-127-3p, we observed evidence for colocalization of signals, but no proxy variant was available.

Huan et al.^15^ reported significant cis-QTL findings for 10 of the 28 miRNAs implicated by our cis-miRNA-QTL associations. A total of 7 of our specific 28 cis-miRNA-QTL associations demonstrated replication (Table 2). The corresponding miRNAs were hsa-miR-181a-5p, hsa-miR-625-3p, hsa-miR-127-3p, hsa-miR-411-3p, hsa-miR-382-5p, hsa-miR-409-3p, and hsa-miR-941. As for the Nikpay et al. data, 24 out of 28 miRNAs were analyzed by Mustafa et al.^22^. For 14 out of these 24 miRNAs, we observed evidence for replication, as reported in Table 2.

Overall, 12 out of the 28 cis-miRNA-QTL associations were not reported in any of the three independent external miRNA-QTL-studies.

### Colocalization analysis with eQTLs

The colocalization analysis with eQTLs was based on Trans-Omics for Precision Medicine (TOPMed) data and identified several top-cis-miRNA-QTLs colocalizing with cis-eQTLs in asthma-related tissues (Table 3). We observed colocalization between the cis-miRNA-QTL statistics for hsa-miR-1908-5p and cis-eQTL statistics for FADS1 in lung tissue, PBMCs, and nasal epithelial cells, and FADS3 in T-cells. Related, the cis-eQTL statistics for TMEM258 colocalized with cis-miRNA-QTL statistics for hsa-miR-1908-5p in monocytes, PBMCs, and T-cells. Furthermore, we detected colocalization between cis-eQTL statistics for MEG3 and cis-miRNA-QTL statistics for multiple miRNAs in the 14q32 cluster in PBMC and whole blood. Next, UCKL1 and hsa-miR-941 cis-statistics colocalized in PBMCs and T-cells. The cis-miRNA QTL signals for hsa-miR-335-3p overlapped with cis-eQTLs for MEST in monocytes, nasal epithelial cells, PBMCs, T-cells, and whole blood. Additional tissue/cell-type-specific colocalizations were observed between SH3TC2 and hsa-miR-584-5p in lung, and MIR100HG/UBASH3B and hsa-miR-100-5p in nasal epithelial cells. All observed eQTL colocalizations are reported in Supplementary Table 3.

**Table 3:** Results of the colocalization analyses between top-cis-miRNA-QTL-associations and eQTLs and sQTLs

miRNA	CHR	eQTL colocalization	sQTL colocalization
hsa-miR-181a-5p	1	–	–
hsa-miR-4433b-3p	2	–	–
hsa-miR-3679-5p	2	–	–
hsa-miR-3131	2	–	NHEJ1 in lung;
hsa-miR-577	4	–	–
hsa-miR-584-5p	5	SH3TC2 in lung;	–
hsa-miR-550a-3-5p	7	–	–
hsa-miR-335-3p	7	MEST in monocytes; MEST in nasal epithelial cells; MEST in PBMC; MEST in T-cells; MEST in whole blood;	-
hsa-miR-873-3p	9	–	–
hsa-miR-873-5p	9	–	–
hsa-miR-1908-5p	11	FADS1 in lung; TMEM258 in monocytes; FADS1 in nasal epithelial cells; TMEM258 in PBMC; FADS1 in PBMC; TMEM258 in T-cells; FADS3 in T-cells;	FADS1 in lung; FADS2 in lung; FADS2 in monocytes; FEN1 in lung; TMEM258 in nasal epithelial cells; TMEM258 in PBMC; TMEM258 in T-cells; TMEM258 in whole blood;
hsa-miR-100-5p	11	MIR100HG in nasal epithelial cells; UBASH3B in nasal epithelial cells;	–
hsa-miR-625-5p	14	–	–
hsa-miR-625-3p	14	LINC02324 in PBMC; LINC02324 in whole blood;	–
hsa-miR-493-5p	14	–	–
hsa-miR-127-3p	14	MEG3 in PBMC; MEG3 in whole blood;	PPP2R5C in PBMC; PPP2R5C in T-cells; PPP2R5C in whole blood;
hsa-miR-432-5p	14	–	–
hsa-miR-136-3p	14	MEG3 in PBMC; MEG3 in whole blood;	–
hsa-miR-370-3p	14	MEG3 in PBMC; MEG3 in whole blood;	–
hsa-miR-411-3p	14	–	–
hsa-miR-654-5p	14	–	–
hsa-miR-382-5p	14	–	–
hsa-miR-323b-3p	14	MEG3 in PBMC; MEG3 in whole blood;	-
hsa-miR-409-5p	14	–	–
hsa-miR-409-3p	14	MEG3 in PBMC; MEG3 in whole blood;	-
hsa-miR-3615	17	–	–
hsa-miR-1-3p	20	–	CRMA in lung;
hsa-miR-941	20	DNAJC5 in nasal epithelial cells; UCKL1 in PBMC; UCKL1 in T-cells;	TPD52L2 in lung; TPD52L2 in T-cells; UCKL1 in whole blood;

*eQTL* expression quantitative trait loci, *sQTL* splicing quantitative trait loci, *PBMC* peripheral blood mononuclear cells, *CHR* Chromosome.

### Colocalization analysis with sQTLs

Investigating the TOPMed splicing QTL results, we observed an overlap between multiple genetic factors underlying miRNAs and sQTLs (Table 3). This includes hsa-miR-1908-5p and FADS1, FADS2, and FEN1 in lung tissue, and TMEM258 in nasal epithelial cells, PBMCs, T-cells, and whole blood. The cis-miRNA-QTL statistics for hsa-miR-127-3p colocalized with sQTLs for PPP2R5C in PBMCs, T-cells, and whole blood. We also observed colocalization between cis-QTLs for hsa-miR-941 and TPD52L2 in lung and T-cells. The cis-miRNA-QTL statistics for the miRNA implicated by the strongest hit in our QTL analysis, hsa-miR-3131, overlapped with sQTL statistics for NHEJ1 in lung tissue. The complete results of the colocalization analysis with sQTLs are reported in Supplementary Table 4.

### Colocalization analysis with GWAS summary statistics

**Fig. 2:**
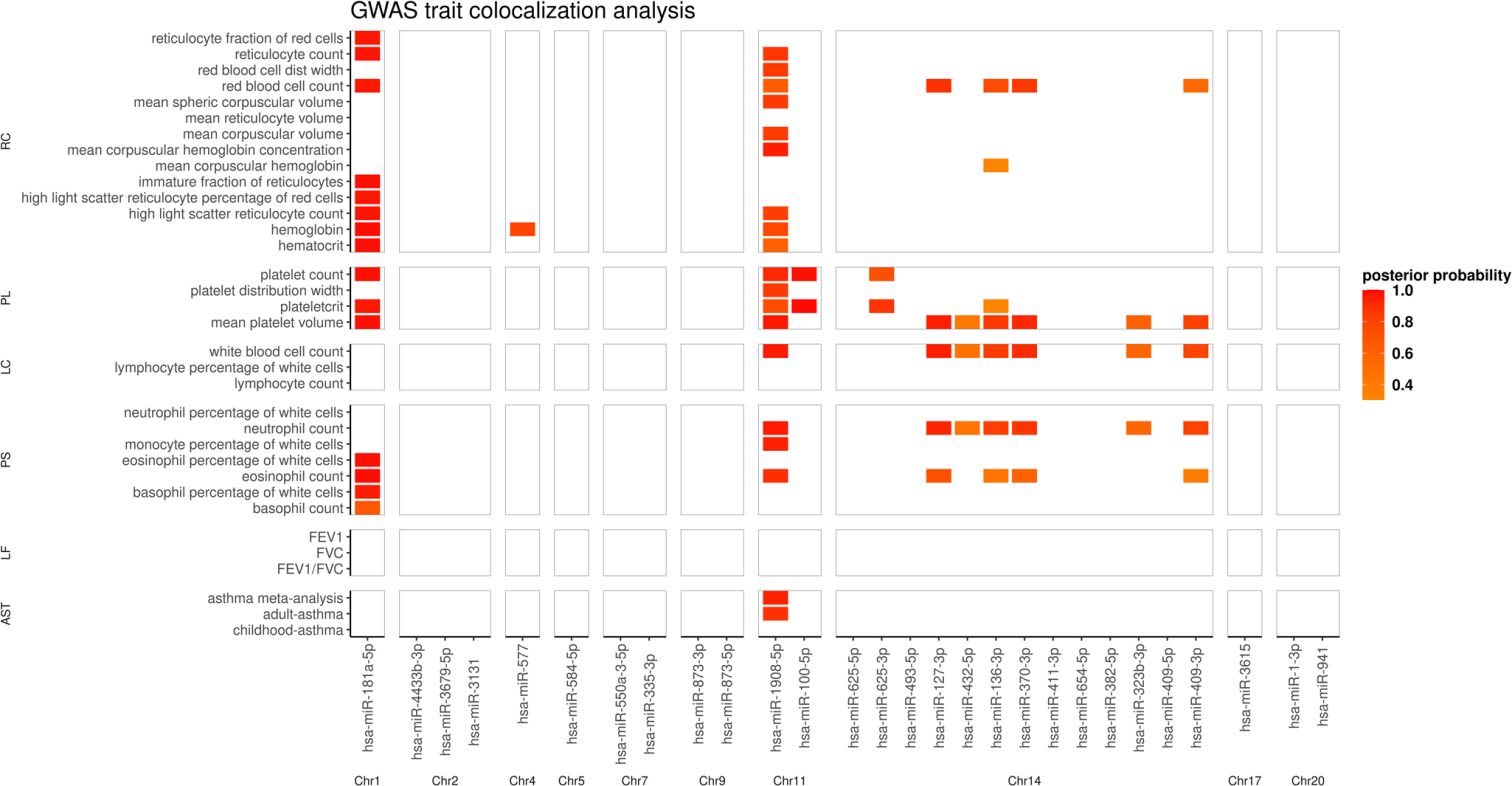
Illustration of the colocalization analysis results between the 28 GACRS top-cis-miRNA-QTL statistics and GWAS summary statistics for asthma-related and blood cell traits. Colored tiles visualize the posterior probability of colocalization. PL: Platelets; LC: Lymphocytes; PS: Phils; LF: Lung function; AST: Asthma; RC: Red cells. This plot was created using the *ggplot2* R package.

We investigated the overlap between the detected cis-miRNA-QTLs and GWAS signals for blood cell traits^23^, lung function^24^, childhood/adult-onset asthma^6^, and a large-scale asthma meta-analysis^5^. Previously, significant associations between blood cell indices and autoimmune diseases such as asthma were observed^25^, including eosinophil counts, neutrophil counts, lymphocyte counts, and monocytes. We obtained the GWAS summary statistics for 28 out of 29 blood cell traits based on European ancestry participants in the UK Biobank^26^, as provided by Vuckovic et al. All traits and the respective results of the colocalization analyses are reported in Supplementary Table 5 and visualized in Fig. 2. The cis-miRNA-QTL statistics for hsa-miR-181a-5p colocalized with the GWAS summary statistics for eosinophil, platelet, and red blood cell counts. Moreover, the hsa-miR-1908-5p cis-QTL signal showed strong colocalization with the summary statistics for neutrophil counts and asthma. The latter was specific to the meta-analysis by Tsuo et al.^5^ and the adult-onset analysis by Ferreira et al.^6^; it was not observed for the corresponding childhood-onset analysis. Plateletcrit GWAS statistics colocalized with the cis-miRNA-QTL statistics for hsa-miR-100-5p and hsa-miR-625-3p. Finally, the cis-QTL statistics for miRNAs in the 14q32 cluster colocalized with several blood cell traits, including neutrophil counts. No colocalizations with lung function GWAS hits were observed.

## Discussion

We performed robust miRNA-QTL analyses using serum miRNA expression levels and whole-genome sequencing data in two studies of childhood asthma. The discovery analysis was based on samples from the Genetic Epidemiology of Asthma in Costa Rica Study, and the results were tested for replication using data from the Childhood Asthma Management Program. We discovered 28 miRNAs with significant top-cis-miRNA-QTLs in GACRS, and four of these top-cis-miRNA-QTL associations were replicated in CAMP. The trans-analysis identified three significant trans-miRNA-QTL associations in GACRS that require further follow-up studies to confirm their validity. Comparing the identified cis-miRNA-QTL associations with the results of three previous relevant miRNA-QTL studies, we observed an overlap of 16 miRNA-QTL-signals, leaving 12 signals that were not reported in these studies. Notably, the cis-QTL association of hsa-miR-3131, with strong replication in CAMP, is novel, to the best of our knowledge.

Previous studies demonstrated that miRNAs are vital biomarkers and research targets for understanding asthma development^10^. Establishing the link between genetic variants and miRNAs, especially in a target population of children with asthma, facilitates this process. Thus, miRNA-QTL associations that potentially appear specific to children (with asthma), such as the signals related to hsa-miR-3131, are of increased interest. Multiple miRNAs linked to the identified cis-miRNA-QTLs were described in prior studies of asthma. In particular, hsa-miR-181a was linked to asthmatic airways and airway remodeling^27^. Moreover, hsa-miR-625, and hsa-miR-409 were linked to childhood asthma^28^. Finally, hsa-miR-370 was demonstrated to be associated with asthma in adults^29^.

Furthermore, we performed extensive colocalization analyses based on the cis-miRNA-QTL statistics and external summary statistics from eQTL, sQTL, and GWAS analyses. The selected eQTL, sQTL, and GWAS summary statistics correspond to asthma-relevant tissues, cells, and traits. We incorporated sQTL associations since RNA splicing potentially plays an important role in the connection between genetic variation and complex diseases^30^. The results showed that cis-miRNA-QTLs overlap with cis-eQTLs and cis-sQTLs of genes, of which multiple were implicated in asthma. These genes include FADS1, FADS2, FADS3, TMEM258, UCKL1, PPP2R5C, TPD52L2, MEG3, and NHEJ1.

FADS1 and FADS2 were linked to asthma in several previous studies^31–34^. For example, Huang et al. reported that an association between polyunsaturated fatty acids and asthma risk was linked to FADS1 polymorphisms^33^. Moreover, Zaied et al. implicated FADS1, FADS2, and TMEM258 in the context of the metabolism of arachidonic acid and overexpression of cysteinyl leukotrienes in patients with asthma^35^. Zhu et al. linked genetic variants in this locus to asthma and fasting glucose^36^. Zhu et al. showed that DNA methylation linked to UCKL1 predicts asthma severity in African American children^37^. Whittle et al. characterized the molecular structure of human dust-mite-associated allergic asthma. The Mitotic Roles of the Polo-Like Kinase pathway was predicted to have significantly altered activity in asthma patients. One of the molecules with decreased gene expression was PPP2R5C^38^. In another study, seropositivity to Ascaris lumbricoides was associated with a CpG site linked to TPD52L2 in men^39^. Chiu et al. detected an association between SNPs in MEG3, asthma status and severity, and MEG3 expression^40^. Furthermore, Feng, Yang, and Yan identified differential MEG3 expression in peripheral blood between individuals with asthma and those without asthma^41^.

The colocalization analysis with the GWAS associations identified strong colocalizations with susceptibility loci for neutrophil, platelet, and eosinophil counts, as well as asthma affection status. These overlaps included hsa-miR-181a-5p, hsa-1908-5p, hsa-miR-100-5p, hsa-miR-625-3p, and the miRNAs located in the 14q32 cluster. Interestingly, this suggests a link between the genetic regulation of miRNAs and blood cell traits, though further analyses are required to disentangle causal relationships. Previous studies established a connection between blood eosinophil counts and asthma-related outcomes^42^.

Our analysis has the unique strength that we assessed the genetic regulation of miRNA expression directly in well-phenotyped children with asthma from populations with high prevalence. Furthermore, GACRS and CAMP collected data based on analogous protocols, and the WGS, as well as miRNA data, were processed using the same procedures.

On the other hand, our study has the following limitations. First, the analyses are based on relatively small sample sizes, limiting both the power of the discovery in GACRS and the replication study in CAMP. Second, although previous work demonstrated the relevance of circulating miRNAs obtained from serum samples in the study of asthma etiology^43^, the functional significance of our findings needs to be assessed in more detail in future studies. This also applies to the hypothesized relevance of the reported novel cis-miRNA-QTL associations in children with asthma based on the absence of these associations in other studies. Since our analyses only include children with asthma, we cannot directly compare these associations in patients with asthma and unaffected individuals. Next, although multiple miRNAs are located on the X-chromosome, our QTL study did not perform a cis-miRNA-QTL analysis for these miRNAs, leaving it as a part of future steps. Moreover, our quality control excluded a small number of genetic variants with highly significant deviations from the Hardy-Weinberg Equilibrium, although these deviations could arise from the multi-ancestry characteristics of the cohorts, especially CAMP, rather than being due to technical artifacts. Additionally, our replication analysis in the three external studies is partly based on nominally significant external summary data only, and does not consider the direction of effect. Lastly, we note that the top-cis-miRNA-QTL analysis did not investigate the existence of independent secondary QTLs in the corresponding region, making the colocalization results sensitive regarding the selected size of the genetic region and potentially reflecting sharing of secondary QTL instead of the primary hit identified in our discovery analysis.

In summary, we characterized genetic determinants of serum miRNA levels in children with asthma using whole-genome sequencing data, discovering novel miRNA-QTL associations that are potentially specific or more common in children with asthma. The underlying genetic factors were linked to asthma-relevant gene expression and splicing regulation, as well as blood cell traits and asthma status GWAS results. This work highlights the connection between genetic factors, miRNAs, and asthma-relevant gene expressions and phenotypes. Future studies will aim to dissect these associations to unravel causal pathways.

## Methods

### Study populations

The miRNA-QTL discovery analysis utilized data from the “Genetic Epidemiology of Asthma in Costa Rica” Study (GACRS). Replication was performed using data from the Childhood Asthma Management Program (CAMP).

GACRS is a cross-sectional cohort of children aged 6 to 14 years of age with asthma (defined as physician-diagnosed asthma and at least two episodes of respiratory symptoms (wheezing, cough, or dyspnea), or a history of asthma attacks in the previous year) and at least six great-grandparents born in the Central Valley of Costa Rica. Recruitment and detailed study descriptions have been discussed in a previous publication^16^.

CAMP was a multi-center, randomized, double-blind clinical trial designed to determine the long-term effects of three inhaled treatments for childhood asthma^17^. CAMP included 1041 ethnically diverse children aged 5 to 12 years old with mild to moderate persistent asthma, as defined by the presence of symptoms or by the use of an inhaled bronchodilator at least twice weekly or the use of daily medication for asthma^44^.

GACRS was approved by the Mass General Brigham Human Research Committee at Brigham and Women’s Hospital (Boston, MA; protocol No. 2000P001130) and the Hospital Nacional de Niños (San José, Costa Rica).

For CAMP, all study procedures were approved by the Mass General Brigham Human Research Committee at Brigham and Women’s Hospital (Boston, MA; protocol No. 2011P000710).

The current work was approved by the Mass General Brigham Human Research Committee at Brigham and Women’s Hospital (Boston, MA; protocol No. 2017P001799). Written parental consent and participating child’s assent were obtained. The authors confirm that the study conforms to recognized standards of the Declaration of Helsinki for medical research involving human subjects. Both studies are registered at https://clinicaltrials.gov/ (NCT00021840: Genetic Epidemiology of Asthma in Costa Rica (first submission 2005-06-23) and NCT00000575: Childhood Asthma Management Program (CAMP) Phases I (Trial), II (CAMPCS), III (CAMPCS/2), and IV (CAMPCS/3) (first submission 2005-06-23)).

### Whole-genome sequencing

Whole-genome sequencing (WGS) data for GACRS and CAMP were generated as part of the National Heart, Lung, and Blood Institute (NHLBI) TOPMed program^45,46^. Details on DNA sample handling, quality control, library construction, clustering and sequencing, read processing, and sequence data quality control are described on the TOPMed website. Our analyses are based on the Freeze 10 release. We extracted bi-allelic autosomal single nucleotide polymorphism (SNPs) and insertions/deletions with a minimum depth of coverage of 10 reads that passed variant quality control (GRCh38 genome reference). We identified a set of unrelated offspring with asthma for the miRNA-QTL analyses using kinship-based inference for GWAS (KING)^47^ and removed sex mismatches and samples with missing rates >=5%. Finally, we excluded genetic variants with a minor allele frequency (MAF) below 5%, Hardy-Weinberg deviation (*p* < 10^-8), or a missing rate >=2%.

### MicroRNA data

Small RNA sequencing (RNA-seq) was performed on serum samples from GACRS and CAMP samples. The details and protocols for this sequencing were described previously^48^, and data was processed in GACRS and CAMP separately. Small RNA-seq libraries were generated based on the Norgen Biotek Small RNA Library Prep Kit (Norgen Biotek, Therold, Canada) and sequenced using the Illumina NextSeq 500 platform by Norgen Biotek. RNA-seq data quality control was performed using the ExceRpt pipeline^49^. MicroRNAs (miRNAs) with less than five mapped reads in 50% of the subjects were removed. Finally, normalization was performed using DESeq2^50^. We utilized miRBase data (version v22)^51–56^ to map miRNAs to (multiple) GRCh38 genomic positions (see Data Availability).

### Cis- and trans-miRNA-QTL analysis

The miRNA-QTL analyses were performed using tensorQTL^18^ and included both cis-(locally) and trans-(at distance)analyses. Discovery analysis was performed using GACRS data, and the results were tested for replication in CAMP data.

#### Cis-miRNA-QTL analysis

The window size parameter to define cis-associations in the tensorQTL cis-analysis was set to the default value of 1 Mb. The cis-analysis performed by tensorQTL identifies the genetic variant with the strongest association with the miRNA expression within the specified genetic cis-region and computes a maximum test statistic and the corresponding *p*-value based on a beta distribution approximation inferred from a permutation distribution. We will refer to this selected genetic variant as the top-cis-miRNA-QTL. The internal empirical *p*-value computations used 10,000 permutations. We included the following covariates in the model: age, sex, batch (in GACRS), and the top five principal components of genetic ancestry (gPCs). Additional sensitivity analyses included the top five or top ten principal components (PCs) of the quality-controlled and normalized miRNA expression count matrix derived from the Reference-Free Adjustment for Cell-Type composition (ReFACTor) approach as covariates^20^ (adopted code, see Code Availability). These additional covariates address the potential presence of unmeasured technical noise or the residual influence of cell type-specific expression profiles across samples with varying cell type proportions. Moreover, we also considered an alternative factor adjustment using the top five and top ten factors from a non-negative matrix factorization of the count matrix. For the non-negative matrix factorization, we utilized the *NMF* R package (see Code Availability). The gPCs were computed from the Genetic Relationship Matrix (via PLINK2) based on genome-wide common variation data for all samples included in the analysis, separately in GACRS and CAMP. Here, the genome-wide genetic variation data were pruned for linkage-disequilibrium (LD) using PLINK2 (command ‘indep-pairwise’ with parameters ‘500 50 0.01’). Significant top-cis-miRNA-QTLs were identified using a False Discovery Rate q < =0.05 (*qvalue* R package) computed based on the top-cis-miRNA-QTL *p*-values across all incorporated miRNA measurements. The replication analysis using CAMP data extracted the variant-miRNA pairs corresponding to the significant top-cis-miRNA-QTLs identified in GACRS and corrected the corresponding nominal association *p*-values in CAMP for the number of tested pairs using a Bonferroni correction. In cases where the top-miRNA-QTL was unavailable in CAMP data, we considered the smallest association *p*-value among strong LD proxies, as identified in GACRS (*r*^2^ > =0.8 in GACRS). Additionally, sensitivity analyses for identified cis-miRNA-QTL associations investigated stratified age analyses (<= 9 years of age vs. > 9 years of age) and ancestry-specific effects by testing interactions between the QTL and gPC1. Finally, we performed a meta-analysis between GACRS and CAMP across all overlapping miRNAs for the cis-miRNA-QTL analysis based on the estimated slopes and corresponding standard errors.

#### Trans-miRNA-QTL analysis

The trans-miRNA-QTL analyses using tensorQTL incorporated the same set of covariates as the cis-analyses. The significance of trans-miRNA-QTL associations was evaluated based on a Bonferroni correction for the number of miRNA measurements tested in GACRS in combination with the genome-wide significance level of $5* {10}^{-8}$, that is $p < \frac{5* {10}^{-8}}{{\#miRNA\; measurements}}$. Significant variant-miRNA pairs were tested for replication in CAMP using the same strategy described for the cis-analysis.

### Replication using results from previously published miRNA-QTL analyses

Nikpay et al. conducted a genome-wide miRNA-QTL analysis of 2,083 miRNAs using plasma samples taken from 710 unrelated subjects of European genetic ancestry^14^. We downloaded the corresponding genome-wide summary statistics (see Data Availability) and tested the cis-miRNA-QTL associations detected in our GACRS analysis for replication. In cases where the top-miRNA-QTL was not available in the data by Nikpay et al., we considered strong LD proxies (criteria r^2^ > =0.8 in GACRS). In addition to evaluating the single variant association data, we performed a colocalization analysis between GACRS and the data by Nikpay et al. for the cis-miRNA-QTL hits. We defined a successful replication by a replication *p*-value that is nominally significant after adjusting for the number of associations tested or a colocalization posterior probability of more than 80%.

We also incorporated the cis-miRNA-QTL results by Huan et al., based on whole blood in 5239 individuals from the Framingham Heart Study^15^. Here, we checked for overlap with our cis-miRNA-QTL findings using the significant results provided by Huan et al. (cis-miRNA-QTL with FDR < 0.1, Supplementary Table 4 in^15^) (see Data Availability), incorporating LD proxies if needed, as described above. A successful replication was defined by a replication *p*-value that is nominally significant after adjusting for the number of associations tested.

Additionally, we included the recent results from genome-wide association studies of 2083 plasma circulating miRNAs in 2178 participants of the Rotterdam Study^22^. Mustafa et al. provided all nominally significant (*p* < =0.05) miRNA-QTL associations. Here, we used the same approach as for the Huan et al. data.

### Colocalization analysis

We performed colocalization analyses to investigate if a) cis-miRNA-QTLs are shared between miRNAs in proximity (GACRS data), b) miRNA-QTL signals are consistent between GACRS and CAMP, c) miRNA-QTLs observed in GACRS are overlapping with previous miRNA-QTL analyses, d) cis-miRNA-QTLs overlap with expression quantitative trait loci (eQTLs) or splicing quantitative trait loci (sQTLs) in asthma-relevant tissues and cells, and e) cis-miRNA-QTLs colocalize with GWAS hits for asthma-related traits. The colocalization analyses were performed using HyPrColoc (*hyprcoloc* R package)^57^, applying default parameters and requiring at least ten overlapping genetic variants. The eQTL and sQTL summary statistics were obtained from the RNA sequencing results (Freeze 1.1) of the TOPMed initiative (see Data Availability). These cis-eQTL and cis-sQTL summary statistics cover nasal epithelial cells, whole blood, lung, monocytes, peripheral blood mononuclear cells (PBMC), and T-cells. GWAS summary statistics were extracted from the analysis of childhood-onset and adulthood-onset asthma^6^, a global meta-analysis of asthma^5^, the meta-analysis of lung function measurements by Shrine et al.^24^, and the UK Biobank-based analysis of 28 blood cell traits by Vuckovic et al.^23^. The latter includes, for example, the analysis of eosinophil counts, lymphocyte counts, and neutrophil counts. Previous studies have shown that blood eosinophil counts and asthma outcomes are related^42^. We declared a detected colocalization using a cutoff of 80% for the colocalization posterior probability. We note that the HyPrColoc analyses between miRNAs within GACRS data face a complete sample overlap.

## Supplementary information

Supplementary Information

Supplementary information

## Data Availability

Whole-genome sequencing data for GACRS and CAMP can be accessed through dbGaP (phs000988 and phs001726, respectively). Additionally, microRNA data can be accessed openly in the Gene Expression Omnibus (GEO):GACRS (Accession: GSE244573) and CAMP (Accession: GSE134897). GWAS summary statistics for the childhood-onset and adulthood-onset asthma analyses by Ferreria et al.^6^ were downloaded from the GWAS catalog^58^ (GCST007800 and GCST007799). The summary statistics for the GWAS of 28 blood cell traits by Vuckovic et al.^23^ were downloaded from the GWAS catalog. We did not include the summary statistics for ‘Monocyte count’, since these summary statistics were not available in the harmonized format (March 2024). The GWAS summary statistics for the multi-ancestry meta-analysis of asthma by Tsuo et al.^5^ were downloaded from https://www.globalbiobankmeta.org/resources. The GWAS summary statistics for FEV1, FVC, and FEV1/FVC by Shrine et al.^24^ were downloaded from the GWAS catalog (GCST007432, GCST007429, and GCST007431). The results of the GACRS and CAMP cis-miRNA-QTL analyses are available at https://www.ncbi.nlm.nih.gov/projects/gap/cgi-bin/study.cgi?study_id=phs001974. The miRNA-QTL summary statistics by Nikpay et al.^14^ were downloaded from https://zenodo.org/records/2560974. The significant miRNQ-QTL associations by Huan et al. were assessed based on the supplementary material for the publication^15^. The nominal significant miRNA-QTL results by Mustafa et al.^22^ were downloaded from https://zenodo.org/records/13869398. The TOPMed cis-e/sQTL results (Freeze 1.1) were generated in a collaboration between the TOPMed Informatics Research Center, TOPMed Multi-Omics working group, and the TOPMed parent studies contributing RNA-seq and distributed to TOPMed investigators. The corresponding summary statistics were made available through TOPMed. Genomic positions (GRCh38) for miRNAs were obtained from miRBase (https://mirbase.org/download/hsa.gff3, v22, downloaded October 2^nd^, 2023). The miRNA expression data from the miRNATissueAtlas2 was downloaded from https://ccb-compute2.cs.uni-saarland.de/mirnatissueatlas_2025/downloads/ (downloaded March 13, 2025).

## Code availability

The ReFACTor approach was adopted from the R code at https://github.com/cozygene/refactor. NMF R package: https://cran.r-project.org/web/packages/NMF/index.html. ggplot2 R package: https://cran.r-project.org/web/packages/ggplot2/index.html. PLINK2 (January 2024): https://www.cog-genomics.org/plink/2.0/. PLINK1.9 (December 2023): https://www.cog-genomics.org/plink/1.9/. R versions 4.1.0 and 4.3.0: https://www.r-project.org/. KING 2.3.2: https://www.kingrelatedness.com/. tensorQTL v1.0.9: https://github.com/broadinstitute/tensorqtl. LocusCompareR R package: https://github.com/boxiangliu/locuscomparer. HyPrColoc R package: https://github.com/cnfoley/hyprcoloc. qvalue R package: https://bioconductor.org/packages/release/bioc/html/qvalue.html. TOPMed website: https://topmed.nhlbi.nih.gov/.
