# Supplementary material for: Serum microRNA expression quantitative trait loci in children with asthma colocalize with asthma-related GWAS results
Source: NPJ Genom Med. 2025 Jul 17;10:55. doi: 10.1038/s41525-025-00510-7 (PMC12271466; doi:10.1038/s41525-025-00510-7)
Supplement: Supplementary file 1 — Supplementary Information [file 41525_2025_510_MOESM1_ESM.pdf]

Supplementary Figures

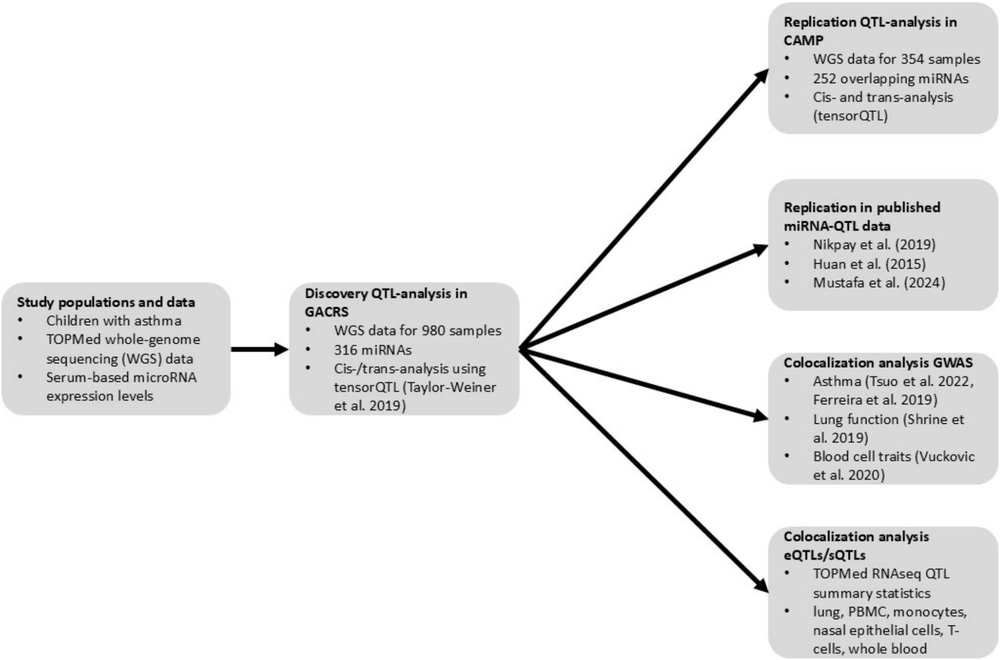

Supplementary Figure 1. Workflow of the miRNA-QTL and downstream analyses.

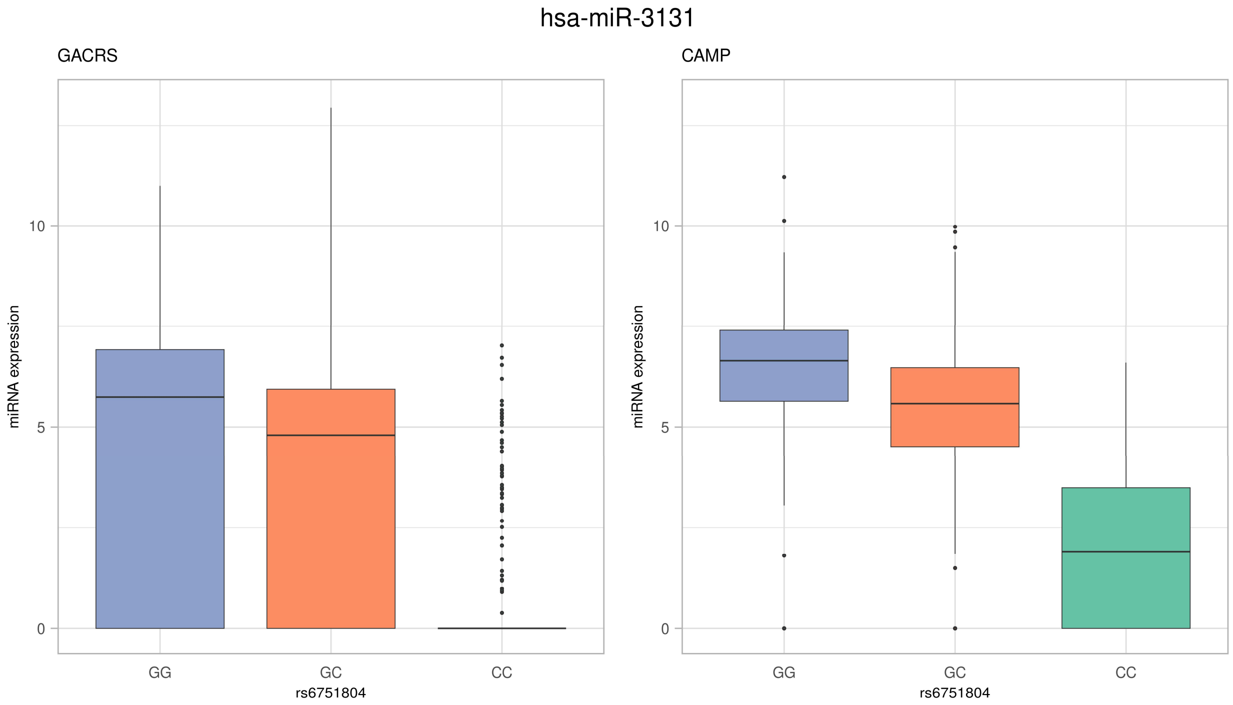

Supplementary Figure 2. Boxplots showing the serum expression levels of hsa-miR-3131 in GACRS and CAMP, stratified by the genotype groups defined by rs6751804.

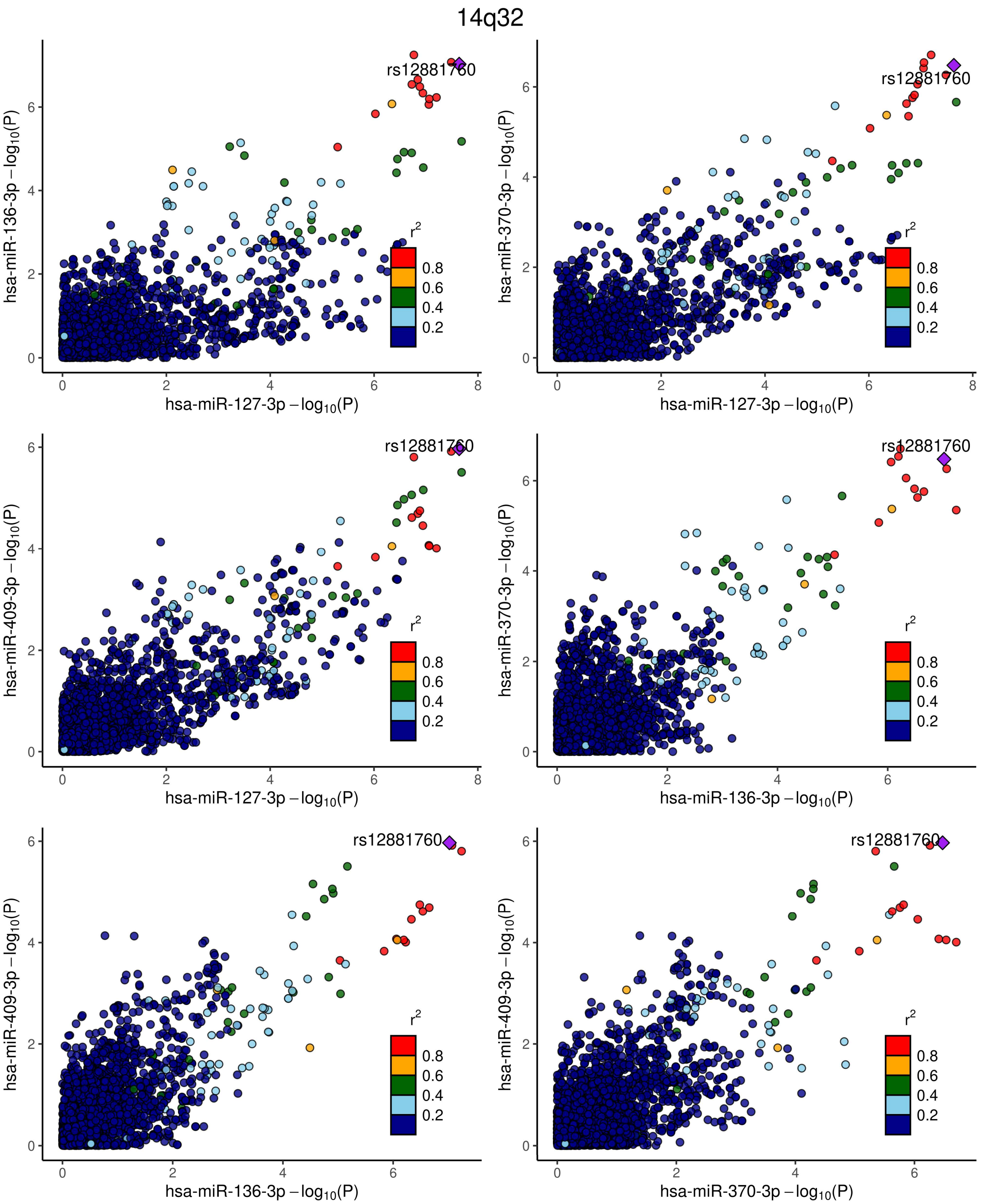

Supplementary Figure 3. Locuscompare plots based on the cis-miRNA-QTL statistics for the four miRNAs hsa-miR-136-3p, hsa-miR-409-3p, hsa-miR-127-3p, and hsa-miR-370-3p in 14q32. The SNP rs12881760 was identified by *HyPrColoc* as the most likely shared genetic factor. This plot was created using the *LocusCompareR* R package.

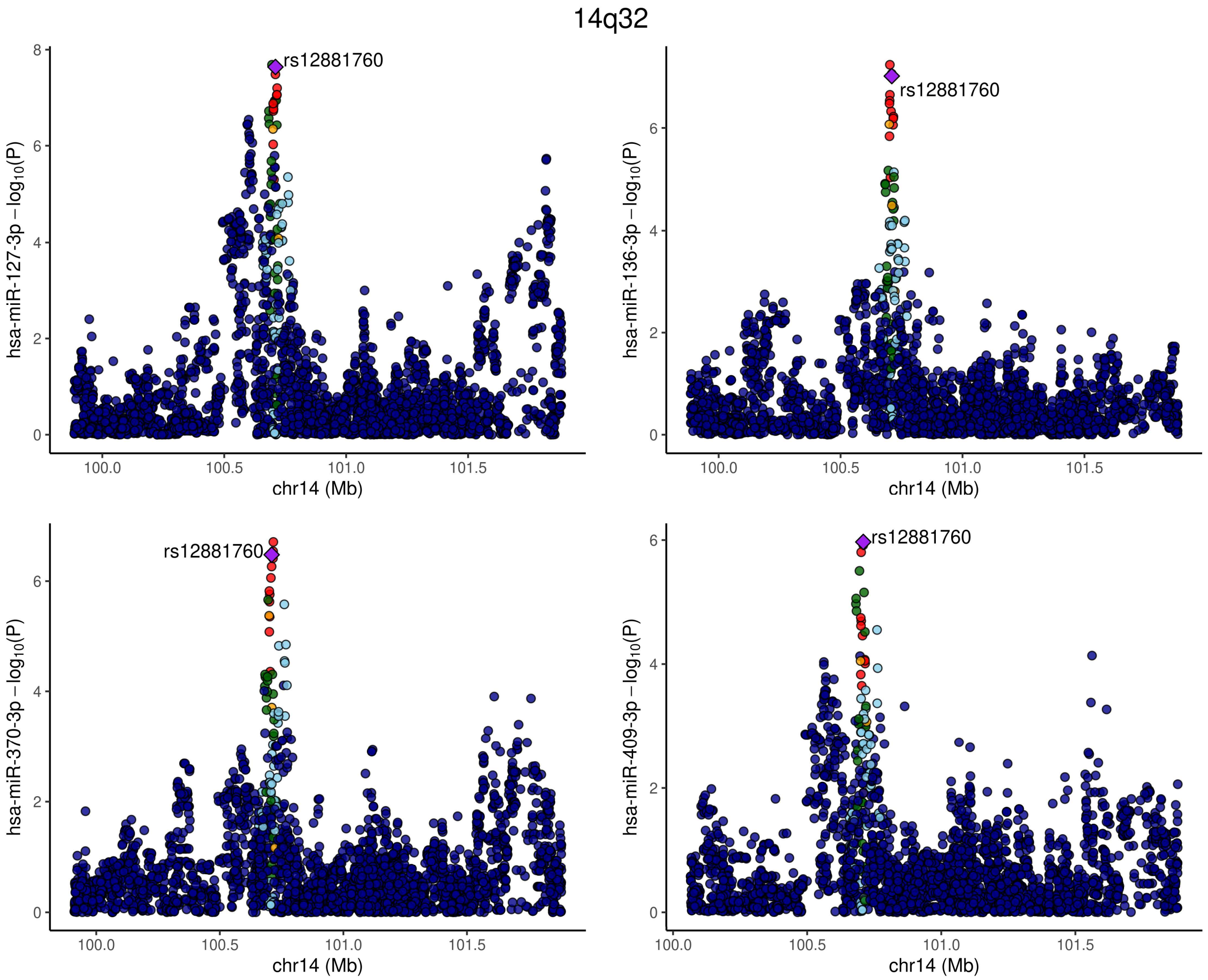

Supplementary Figure 4. Locuszoom plots based on the cis-miRNA-QTL statistics for the four miRNAs hsa-miR-136-3p, hsa-miR-409-3p, hsa-miR-127-3p, and hsa-miR-370-3p in 14q32. The SNP rs12881760 was identified by *HyPrColoc* as the most likely shared genetic factor. This plot was created using the *LocusCompareR* R package.
